# Supplementary material for: Basis of specificity for a conserved and promiscuous chromatin remodeling protein
Source: eLife. 2021 Feb 12;10:e64061. doi: 10.7554/eLife.64061 (PMC7968928; doi:10.7554/eLife.64061)
Supplement: Supplementary file 1. [file elife-64061-supp1.docx]

| **Strain** | **Genotype** | **Source** |
| --- | --- | --- |
| YS001* | W303 MATa ade2-1 can1-100 his3-11,15 leu2-3, 112trp1-1 ura3-1 RAD5+ | Tsukiyama strain YTT0116 |
| YS009 | W303 MATa UME6-3xFLAG::KANMX | Tsukiyama strain YTT691 |
| YS488 | W303 MATa ISW2(K215R)-3xFLAG::KANMX | This Study |
| YS517 | W303 MATa ITC1[aa1-73]-3C-3xFLAG-SpyTag002::HPHMX | This Study |
| YS518 | W303 MATa ITC1[aa1-132]-3C-3xFLAG-SpyTag002::HPHMX | This Study |
| YS778 | W303 MATa ume6∆::NATMX, [pRS416 (Ume6p**_Ume6[764-836]DBD-3xFLAG-SpyTag002), CEN, | This Study |
| YS242 | W303 MATa UME6[∆aa2-479] | This Study |
| YS828 | W303 MATa UME6[∆aa480-508] | This Study |
| YS831 | W303 MATa rpd3∆::hphMX | This Study |
| YS816 | W303 MATa UME6[∆aa2-479], rpd3∆::hphMX | This Study |
| YS812 | W303 MATa UME6[∆aa2-508], rpd3∆::hphMX | This Study |
| YS142 | W303 MATa UME6[∆aa2-97] | This Study |
| YS144 | W303 MATa UME6[∆aa2-175] | This Study |
| YS146 | W303 MATa UME6[∆aa2-322] | This Study |
| YS243 | W303 MATa UME6[∆aa2-479] | This Study |
| YS147 | W303 MATa UME6[∆aa2-508] | This Study |
| YS148 | W303 MATa UME6[∆aa2-596] | This Study |
| YS828 | W303 MATa UME6[∆aa480-508] | This Study |
| YS496 | W303 MATa UME6[∆aa2-596], [pRS416 (ADH_Ume6[480-508]-SpyCatcher), CEN, URA3] | This Study |
| YS313 | W303 MATa isw2∆::KANMX, isw1∆::HPHMX | This Study |
| YS312 | W303 MATa isw2∆::KANMX, chd1∆::HPHMX | This Study |
| YS322 | W303 MATa isw1∆::KANMX, chd1∆::NATMX | This Study |
| YS335 | W303 MATa isw1∆::KAN, chd1∆::NAT, isw2∆::HPH | This Study |
| YS336 | W303 MATa isw1∆::KAN, isw2∆::NAT, chd1∆::HPH | This Study |
| YS045 | W303 MATa isw2∆::URA3 | This Study |
| YS385 | W303 MATa mbp1∆::HPHMX | This Study |
| YS642 | W303 MATa swi4∆::NATMX | This Study |
| YS494 | W303 MATa swi6∆::NATMX | This Study |
| YS453 | W303 MATa MBP1[aa1-136]-SpyTag-3xFLAG::NATMX | This Study |
| YS590 | W303 MATa MBP1[aa1-136]-SpyTag-3xFLAG::NATMX, [pRS416 (ADH_Ume6[480-508]-SpyCatche | This Study |
| YS276 | W303 MATa ITC1[∆aa24-130] | This Study |
| YS227 | W303 MATa ITC1[∆aa22-200] | This Study |
| YS229 | W303 MATa ITC1[∆aa22-273] | This Study |
| YS098 | W303 MATa ITC1[∆aa9-374] | This Study |
| YS895 | W303 MATa ITC1(E33R, E40H) | This Study |
| YS897 | W303 MATa ITC1(E43R, D70K) | This Study |
| YS892 | W303 MATa ITC1(E33R, E40H, E43R, D70K) | This Study |
| YS246 | W303 MATa UME6[Δaa2-479]-3xFLAG::KANMX | This Study |
| YS260 | W303 MATa UME6[Δaa2-508]-3xFLAG::KANMX | This Study |
| YS458 | W303 MATa MBP1[aa1-561]-SpyTag-3xFLAG::NATMX | This Study |
|  |  |  |
| *all strains used in this study are derived from this background; for brevity, this genotype is indicated by “W303 MATa” in subsequent strains | | |
| **Ume6p = endogenous Ume6 promoter | |  |
