# Supplementary material for: Basis of specificity for a conserved and promiscuous chromatin remodeling protein
Source: eLife. 2021 Feb 12;10:e64061. doi: 10.7554/eLife.64061 (PMC7968928; doi:10.7554/eLife.64061)
Supplement: Supplementary file 2. [file elife-64061-supp2.docx]

| **Plasmid** | **Use** | **Source** |
| --- | --- | --- |
| P001 | pUG 6 (knockouts with KANMX) | Tsukiyama plasmid 1 |
| P003 | P2L-3FLAG-KANMX | Tsukiyama plasmid 251 |
| P002 | pAG 32 (knockouts with HPHMX) | Tsukiyama plasmid 3 |
| P016 | pAG 25 (knockouts with NATMX) | Tsukiyama plasmid 251 |
| P143 | 3xFLAG-SpyTag::NATMX | Donovan, *et al*27 |
| P259 | pRS416_ADH_Ume6[480-508]_SpyCatcher, CEN, URA | This Study |
| P300 | pRS416_Ume6p*_Ume6[764-836]_3xFLAG_SpyTag002, CEN | This Study |
|  |  |  |
| *Ume6p = endogenous Ume6 promoter | |  |
